# Selective Adsorption, Reduction, and Separation of Au(III) from Aqueous Solution with Amine-Type Non-Woven Fabric Adsorbents

**DOI:** 10.3390/ma13132958

**Published:** 2020-07-02

**Authors:** Chen Huang, Xiao Xu, Junxuan Ao, Lin Ma, Feng Ye, Ziqiang Wang, Lu Xu, Xiaoyan Zhao, Hongjuan Ma

**Affiliations:** 1 School of Petrochemical Engineering, Changzhou University, Changzhou 213164, China; huangchen@sinap.ac.cn; 2 Shanghai Institute of Applied Physics, Chinese Academy of Sciences, Shanghai 201800, China; xuxiao@sinap.ac.cn (X.X.); aojunxuan@sinap.ac.cn (J.A.); malin@sinap.ac.cn (L.M.); yefeng@sinap.ac.cn (F.Y.); wangziqiang@sinap.ac.cn (Z.W.); xulu@sinap.ac.cn (L.X.); 3 University of Chinese Academy of Sciences, Beijing 100049, China

**Keywords:** irradiation, graft polymerization, Au(III), adsorption, selectivity, reduction

## Abstract

Herein, adsorption, separation, and reduction of Au(III) from its aqueous solution were studied with different amine-type, non-woven fabric (NF) adsorbents fabricated with radiation-induced graft polymerization. The adsorbents exhibited different adsorption capacities of Au(III) over a concentration range of hydrochloric acid (HCl) from 5 mM to 5 M, and the diethylamine (DEA)-type adsorbent performed best under all test conditions. The DEA-type adsorbent was inert toward other metal ions, including Cu(II), Pb(II), Ni(II), Zn(II) and Li(I), within the fixed concentration range of HCl. Flow-through adsorption tests indicated DEA-type adsorbent exhibited a rapid recovery and high adsorption capacity of 3.23 mmol/g. Meanwhile, DEA-type adsorbent also exhibited high selectivity and rapid extraction for Au(III) from its mixed solution with Pt(IV) and Pd(II). After adsorption, the reduction of Au(III) was confirmed by XRD spectra, TEM, and digital micrograph images. The results indicated that nano-sized Au particles were mainly concentrated on the adsorbent in 5 mM HCl solution. In 1 M HCl solution, not only nano-sized Au particles were found, but also micro-size Au plates precipitation occurred. This study provides a novel material for selective and efficient gold uptake from aqueous solution.

## 1. Introduction

Gold has widely been used because of its excellent physical and chemical properties [1,2]. Meanwhile, gold is also an essential element in electronic communications and the aerospace and medical industries [3,4,5]. However, gold resources on Earth are small and non-renewable, while at the same time, there is increasing demand and decreasing amounts of gold deposits which will limit the practical application of gold to a certain extent, such that the recovery and reuse of gold is of particular importance. For this reason, precious metals in wastewater have gradually attracted individual’s attention [6,7]. Because recycling from aqueous solution is more economical than mining and metallurgy, and it is simple to operate and environment friendly [8,9,10].

In recent years, methods such as co-precipitation [11], adsorption [12,13,14,15,16], electrochemical [17], bioleaching and solvent extraction [18,19], etc., have been used to extract precious metals from solutions. Among these methods, adsorption is considered to be a hopeful method, given the advantages of high efficiency, simple operation, and lower wastes discharge [12,13,14,15,16]. Much research has been conducted on the recovery of gold in precious metal solutions. Traditional adsorbents, such as activated carbon, has the advantages of low cost and developed porosity. However, it only relies on physical adsorption, it is not easy to regenerate, and it lacks a reducing ability which has certain limitations for the recovery of Au [20,21]. Adsorption resin is also a common research object with a higher adsorption rate but poor selectivity [8,22]. Biosorbent, although rich in specific functional groups, the stability and selectivity in solution needs to be further improved [15,23]. Therefore, it is of considerable significance to study adsorbents with good selectivity, stability, and reducibility to recover gold from solution.

In this work, recovery of Au(III) using various kinds of amine functionality adsorbent with different β-side-chain length synthesized via radiation-induced emulsion graft polymerization was studied with both batch and column models. Sorption isotherm, sorption kinetics, sorption capacity of Au(III) and the effect of HCl concentration in solution were investigated. The best amine-type adsorbent (DEA-type) in this study were inert toward not only basic metals including Cu(II), Pb(II), Ni(II), Zn(II), and Li(I) within the fixed concentration range of HCl but also other precious metals of Pt(IV) and Pd(II), which exhibited high selectivity to Au(III). In addition, the reduction of gold was also explored via TEM and digital micrograph images. The results revealed the formation of nano-sized Au particles and micro-sized Au plates in different conditions. The adsorption, separation, and reduction process of Au(III) by amine-type adsorbent synthesized via radiation-induced emulsion graft polymerization are presented, and the results provide a novel material for selective and enhanced gold recovering from aqueous solution.

## 2. Materials and Methods

### 2.1. Materials

Polyethene-coated polypropylene (PE/PP) non-woven fabric (NF) was purchased via Kurashiki MFG Company (Osaka, Japan) and used as the substrate polymer. 4-Hydroxybutyl acrylate glycidyl ether (4-HB) obtained from Tokyo Kasei Kogyo Co. Ltd. (Tokyo, Japan), was used without further purification. Ethylenediamine (EDA), diethylenetriamine (DETA), triethylenetetramine (TETA), diethylamine (DEA), tris(2-aminoethyl)amine (TAEA), 2,4-diamino-6-butylamino-1,3,5-triazine (DABA), surfactant sorbitan monolaurate (Span-20), xylene, and isopropyl alcohol (IPA) were obtained via Kanto Chemical Co., Ltd. Analytical grade HAuCl_4_·4H_2_O was used to prepare Au(III) solutions. Standard solution with the concentration of 1000 ppm were used for the preparation of other metal solutions. Analytical grade hydrochloric acid (HCl) was used without further purification. Other solvents of reagent grade, including methanol (MeOH), were for washing treatment.

### 2.2. Preparation of Amine-Type Adsorbent

**Scheme 1:**
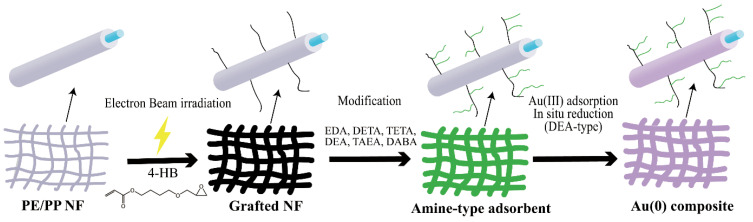
Schematic diagram of the preparation for amine-type adsorbents.

The adsorbent was synthesized based on the radiation-induced graft polymerization outlined in Scheme 1. Non-woven fabric were cut into squares and enclosed into polyethylene bags. After deoxygenation by purging N_2_ gas, the NF pieces were exposed by electron beam (EB) irradiation with the absorbed dose of 30 kGy. Then, the NF pieces were placed in a glass ampoule, and high purity nitrogen was added to remove excess air. An emulsion with 5% 4-HB and 0.5% surfactant Span-20 was drawn into the glass ampoule by suction [24]. The glass ampoule was kept in a water bath with a temperature of 40 °C. After 2 h, the samples were taken out and rinsed with methanol three times and dried in a vacuum oven (Shanghai Chemical Reagent Co., Ltd., Shanghai, China). The obtained 4-HB-grafted NFs with 150% degree of grafting (Dg) were modified by organic amine (EDA, DETA, TETA, TAEA, DABA, and DEA) as following: the 4-HB-NFs were immersed in 70% of EDA, DETA, TETA, and TAEA in IPA solution at 60 °C for 4 h, after modification the obtained samples were referred to EDA-type adsorbent, DETA-type adsorbent, TETA-type adsorbent, and TAEA-type adsorbent, respectively. The modification with DEA was carried out for 5 h in a water solution containing 50% DEA at 30 °C, then obtained DEA-type adsorbent. In the case of DABA, grafted NF was immersed in a saturated DABA IPA solution (about 3% DABA) at 60 °C for 8 h, the modified sample was called DABA-type adsorbent. Subsequently, the fibers were washed by distilled water three times than dried in a vacuum oven. The density of the amine group on the samples was calculated as before [24,25].

(1) $$\mathrm{Amine}\;\mathrm{group}\;\mathrm{density}\;\left(\mathrm{mmol}/g\right)=\frac{W_{i}{-W}_{0}}{{M\;\times \;W}_{i}}\;\times \;1000$$

where W_0_ and W_i_ referred to the weights of the samples before and after modification. M is the molecular weight of the amine compound. The detail of the functional groups in different adsorbents are listed in Table 1.

### 2.3. Characterization

The surface sections of NFs were characterized via a scanning electron microscope (Hitachi SEMEDX Type-N). The grafting of 4-HB on PE/PP, modified with amine groups and adsorption performance were demonstrated by Fourier transform infrared (PE-Frontier, Perkin–Elmer, MA, USA) and X-ray photoelectron spectroscopy (Nexsa, Thermo Fisher Scientific, MA, USA). Morphology of gold crystal was carried out by a microscope (Keyence VHX Digital). Transmission electron microscopy (FEI TECNAI G2 TEM) and X-ray diffraction (XRD) was used to characterize the morphology of gold particles.

### 2.4. Adsorption and Elution, Column and Batch Mode

Adsorption experiments of the resulting samples were conducted in both batch and column sorption tests. In the kinetics experiment, 0.01 g of adsorbent was added to 10 mL of Au(III) solution. The mixture was stirred continuously at 15 °C. Aliquots of 0.5 mL solution were withdrawn at a specific time, and the concentration of metal ion was determined by an optical emission spectrometer (Optima 4300 DV). The removal ratio (R) of metal ions was calculated as follows:

(2) $$\mathrm{Removal}\;\mathrm{Ratio}\;\left(R\right)\left(\%\right)=\frac{C_{0}\;-\;C}{C_{0}}\;\times \;100$$

where C_0_ and C are the initial concentrations and the concentration after adsorption, respectively. The adsorption capacities (Q, mmol g^−1^) were evaluated using the following equation:

(3) $$Q=\frac{C_{0}\;-\;C}{V\;\times \;W}$$

where V represents the volume of Au(III) solution (ml), and W is the weight of dry adsorbents (g).

The isothermal adsorption experiments were also performed in batch tests. The samples of 0.01 g were shaken in 10 mL solutions for 2 h at 15 °C with different metal ions concentrations. The concentration of HCl was fixed at 1 M.

To investigate the effect of HCl, 0.01 g samples were added in 10 mL Au(III) solutions, which concentration was 0.5 mM, then adjusted concentrations of HCl in the range from 0.5 mM to 5 M and heated to 15 °C for 2 h.

In the case of column adsorption experiments, the samples were cut into pieces with a diameter of 7 mm and then fixed in a column. Then, experiments of breakthrough and elution were conducted in a PSM071AA model peristaltic pump. A feed solution with metal ions was pumped through the column at a fixed flow rate. The effluent solution was collected at specific time intervals by a programmable fraction collector (CHF122SC model). In the elution experiment, a solution containing 0.01 M HCl and 0.2 M thiourea was used. Thiourea is a reductive organic complexing agent that can react with gold to form soluble complex ions under acidic conditions [26]. The optical emission spectrometer (Optima 4300 DV, Perkin–Elmer, MA, USA) determined the concentrations. The breakthrough point was defined as feeding volumes at the concentration ratio of effluent to pumping solution C/C_0_ up to 0.05, where C_0_ and C referred to the concentrations of metal ions in the feeding solution and in the column effluent, respectively.

## 3. Results and Discussion

### 3.1. Characterization of the Adsorbent

**Figure 1:**
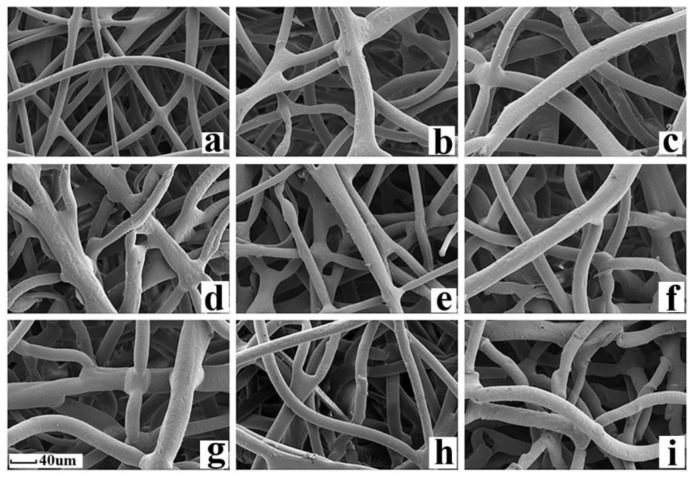
SEM images from the surface of (**a**) 4-HB grafted NF; (**b**) modified NF by EDA; (**c**) DETA; (**d**) TETA; (**e**) DEA; (**f**) TAEA; and (**g**) DABA; (**h**) the surface of EDA; and (**i**) DEA-type adsorbent after elution of Au(III).

Exploration of process conditions for the graft and modification has been completed in previous work [24,27,28]. After graft polymerization of 4-HB onto PE/PP NF, several organic amines were used to modify the material into functional adsorbents, and the morphology of them were studied by SEM, the micrographs equal in scale. The 4-HB grafted NF revealed the fiber morphology, crisscrossed by a network of fibers with 20 µm in diameter and the surface was smooth (Figure 1a). After modification by amine compounds, asperities and swell were observed, while no trail of destruction was observed, and the diameter was between 20 µm and 50 µm with dense surfaces (Figure 1b–g). Combining Table 1 and Figure 1b–g, it is clear that there is no specific relationship between the functional density and the fiber diameter of the adsorbent. Diameters of the EDA-type (Figure 1b), DETA-type (Figure 1c), and TETA-type (Figure 1d) adsorbents were smaller than that of the DEA-type (Figure 1e), TAEA-type (Figure 1f), and DABA-type (Figure 1g) adsorbents as the former three types were linear chain amines and the latter three were branched-chain amines.

**Figure 2:**
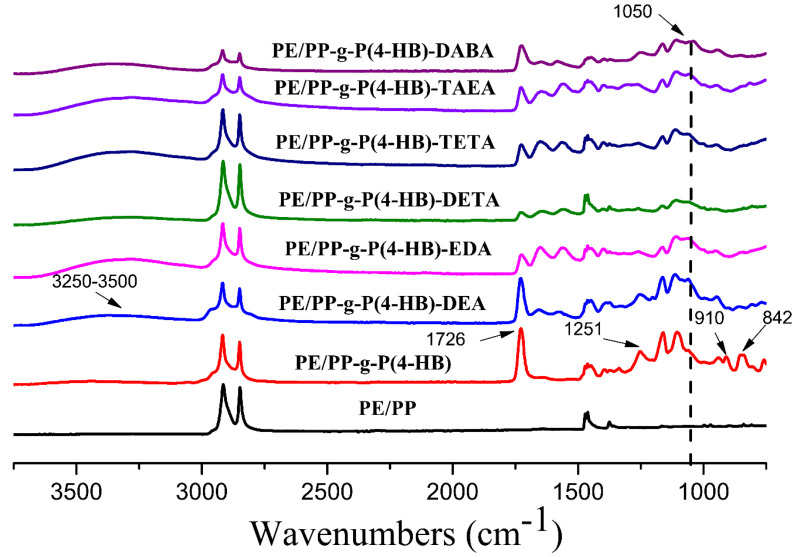
FTIR spectra of trunk PE/PP nonwoven fabric, PE/PP-g-P(4-HB) and modified PE/PP-g-P(4-HB) with DEA, EDA, DETA, TETA, TAEA and DABA.

The FTIR spectra of the trunk PE/PP-grafted materials (denoted as PE/PP-g-P(4-HB)), modified materials (denoted as PE/PP-g-P(4-HB)-DEA, PE/PP-g-P(4-HB)-EDA, PE/PP-g-P(4-HB)-DETA, PE/PP-g-P(4-HB)-TETA, PE/PP-g-P(4-HB)-TAEA and PE/PP-g-P(4-HB)-DABA, respectively) are shown in Figure 2. Compared with the spectrum of PE/PP, the spectrum of the PE/PP-g-P(4-HB) reveals characteristic peaks at 1726, 1251, 910, and 842 cm^−1^, which was assigned to C=O stretching, C–O stretching and characteristic vibrations of epoxy groups, indicated that the 4-HB was grafted onto the trunk PE/PP. After the reaction of the epoxy groups with DEA, EDA, DETA, TETA, TAEA, and DABA, respectively. The characteristic peak at ~1050 cm^−1^ represents the C–N stretching vibration [29], and the absorption bands attributed to characteristic vibrations of epoxy groups at 910, and 842 cm^−1^ disappeared. Moreover, the broadband at ~3400 cm^−1^ was attributed to –OH stretching. Confirming that the reaction between the organic amine and epoxide group was anchored on the PE/PP.

**Figure 3:**
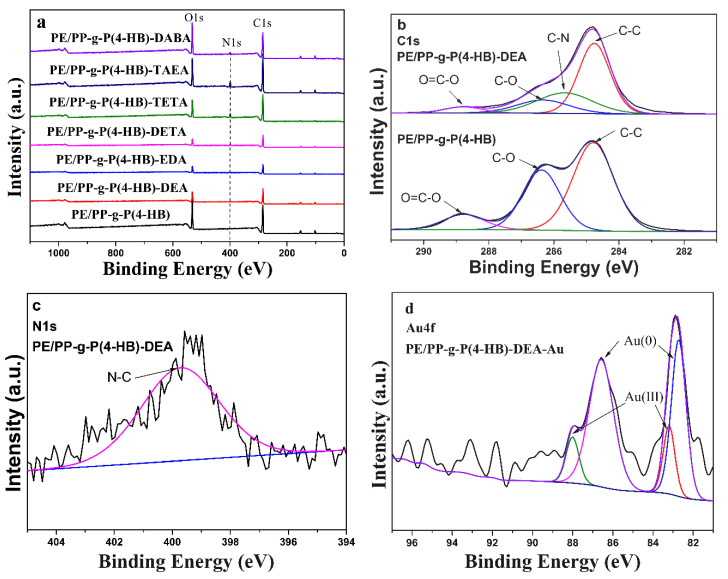
The XPS wide-scan spectra of (**a**) PE/PP-g-P(4-HB) and modified PE/PP-g-P(4-HB) with DEA, EDA, DETA, TETA, TAEA, and DABA; (**b**) C 1s spectra of PE/PP-g-P(4-HB) and modified PE/PP-g-P(4-HB) with DEA; (**c**) N1s spectra of PE/PP-g-P(4-HB)-DEA; and (**d**) Au4f spectra of PE/PP-g-P(4-HB)-DEA-Au.

The XPS analysis was also used to characterize the synthesis of materials. As shown in Figure 3a. The strong peak at BE of 530.1 eV is referring to O1s, which belongs to 4-HB. After PE/PP-g-P(4-HB) ring-opening reaction with the organic amine, a new signal appeared at BE of 400.6 eV (N1s) means the successful modification of amino group. In Figure 3b, take DEA as an example, according to the C1s core-level spectra of PE/PP fiber before and after modification with DEA. The peaks of O=C–O, C–O, and C–C that are part of the general chemical structure of PE/PP-g-P(4-HB) were identified at 288.6 eV, 286.4 eV, and 284.8 eV, respectively [30]. After modified with DEA, a new peak for C–N appeared at BE of 285.58 eV [31]. Furthermore, as shown in Figure 3c, to further verify the successful modification of DEA, the N1s core-level spectrum of PE/PP-g-P(4-HB)-DEA was decomposed into one peak at BE of 400.58 eV attributed to N–C [32]. Those results reveal that PE/PP-g-P(4-HB)-DEA fibers are successfully synthesized, which might provide available binding sites for Au(III) adsorption. In Figure 3d, after adsorption of Au(III), two new signals appeared at BE of 86.8 eV and 82.95 eV possible due to Au4f5/2 and Au4f7/2. The Au4f spectrum can be fitted into four peaks, two of them at BE of 82.88eV and 86.67 eV were from A(0) and the other at BE of 83.55 eV and 87.17 eV were attributed to A(III) [31]. Suggesting that Au(III) was successfully adsorbed and partially reduced.

### 3.2. Sorption Kinetics and Sorption Isotherm of Au(III)

**Figure 4:**
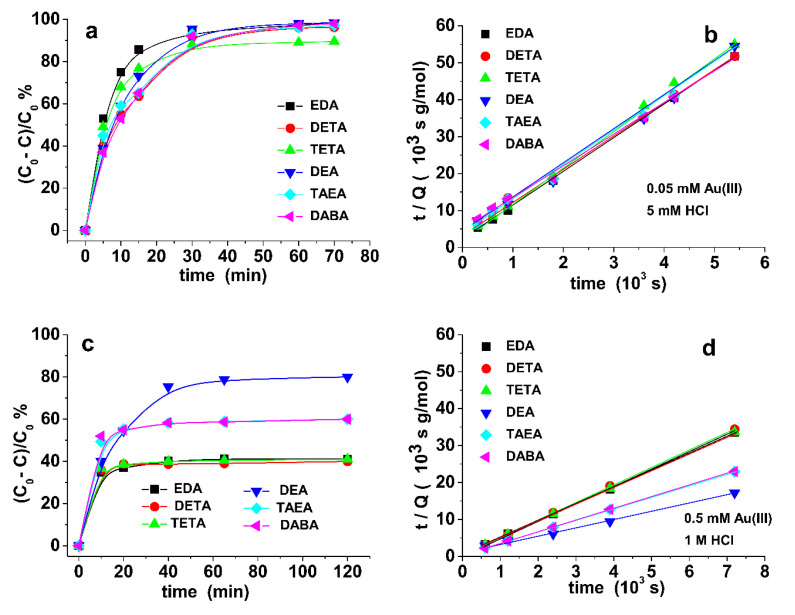
(**a**,**c**) Removal ratio of Au(III) and (**b**,**d**) pseudo-second-order model of Au(III) with initial concentration of 0.05 mM at 5 mM HCl and 0.5 mM at 1 M HCl and obtained from various amine-type adsorbent.

Sorption kinetics was performed with different initial concentrations of Au(III) and HCl in solution. Results from Figure 4 indicated that adsorption equilibrium was obtained within 1 h from these amine-type adsorbents. As shown in Figure 4a, removal ratio of 0.05 mM Au(III) from solution with 5 mM HCl increased along with contact time and reached 98% with the EDA, DETA, DEA, TAEA, and DABA-type adsorbents, while TETA-type adsorbents exhibited only 89%. In the case of higher concentration of Au(III) (0.5 mM) and HCl (1 M) in solution the removal ratio was significantly reduced (Figure 4c). The highest removal ratio was 79% with DEA-type adsorbent, while the EDA and DABA types were 41% and 59%, respectively.

The adsorption kinetics were fitted and calculated by adsorption model with different orders. Results from Figure 4b,d indicated that for each condition, the points matched well with the pseudo-second-order kinetic model (Equation (4)) because of a high correlation coefficient (*R*^2^ > 0.999).

(4) $$\frac{t}{Q}=\frac{1}{k_{2}Q_{e}^{2}}+\frac{t}{Q_{e}}$$

where Q_e_ is the equilibrium adsorption capacity (mmol g^−1^); t referred to the adsorption time (s). k_2_ is the values of rate constants and tabulated in Table 2. Conclusions were drawn from data in Figure 4 and Table 1. EDA-type adsorbent showed the highest constant of adsorption rate for Au(III). For the EDA-, DETA-, TETA-type adsorbents, the orders of rate constants did not exactly agree with the order of β-side-chain length of polymer TETA > DETA > EDA. However, adsorption rate constants were higher than that obtained from the DEA-type adsorbent. Two possible explanations for this were: (a) the grafted functional groups located in the β-side-chain from EDA, DETA, and TETA modified material were longer and more flexible compared with DEA-type adsorbent; (b) the steric effect of methyl groups of DEA impeded the ion-exchange rate (Table 1). DEA-type adsorbent showed the lowest t/Q, which means the highest Q in each condition. According to the information in Table 1, protonation of the amino groups in the adsorbents resulted in various type of ammonium salts and enhanced the electrostatic attraction between anionic AuCl_4_^−^ and the protonated amino groups [31]. Hence, DEA-type exhibited the highest ion-exchange capacity of Au(III). The DABA-type adsorbent also showed remarkable adsorption capacity of Au(III) because of the abundant secondary, tertiary, and quaternary ammonium. The ion-exchange capacity was higher than EDA-, DETA-, and TETA-type adsorbents even with an order of magnitude lower functional group density of 0.2 mmol/g. However, the high price and poor solubility of DABA hampered its application.

**Figure 5:**
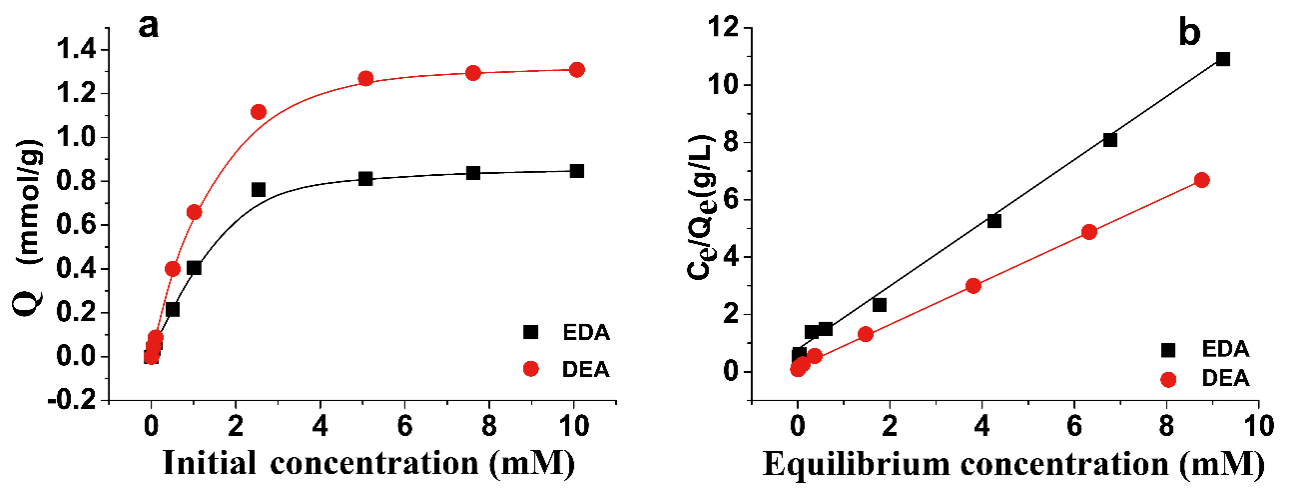
(**a**) Effect of initial concentration on adsorption capacity and (**b**) modeling by the Langmuir model.

The adsorption isotherms for Au(III) by EDA- and DEA-type adsorbent were investigated at 1 M HCl condition. The adsorption isotherm is a curve of the relationship between the concentration of the adsorbate at a specific temperature and the cumulative adsorption amount on the surface of the adsorbent, which can partially reflect the mechanism of the adsorption process. Langmuir isotherm model and Freundlich isotherm model were selected to fit the adsorption data. The Langmuir isotherm equation and its linear form are presented as Equations (5) and (6), the Freundlich isotherm equation and its linear form are presented as Equations (7) and (8), respectively.

(5) $$Q_{e}=\frac{Q_{\max}K_{L}C_{e}}{{1+K}_{L}C_{e}}$$

(6) $$\frac{C_{e}}{Q_{e}}=\frac{C_{e}}{Q_{\max}}+\frac{1}{Q_{\max}K_{L}}$$

(7) $$Q_{e}{=K}_{F}C_{e}^{\frac{1}{n}}$$

(8) $${\mathrm{logQ}}_{e}{=\mathrm{logK}}_{F}\;+\;(\frac{1}{n}{)\mathrm{logC}}_{e}$$

where Q_e_ is the equilibrium adsorption capacity (mmol g^−1^), C_e_ is the equilibrium concentration of Au(III) ions (mmol/L). Q_max_ is the maximum adsorption capacity. K_L_ represents the Langmuir constant connection with the energy of adsorption and K_F_ is the Freundlich constant. The relationship between Q_e_ and C_e_ is shown in Figure 5b. For both EDA and DEA-type adsorbent, the adsorption capacity increased with the increment of the initial concentration of Au(III) until the region of saturation (Figure 5a). The Langmuir model is better to fit the isotherm curve of these two amine-type adsorbents for Au(III) because higher correlation coefficient was obtained in Langmuir model. As shown in Figure 5b and Table 3, the correlation of the Langmuir isotherm model is better (R^2^ > 0.99). Meanwhile, after calculation, the maximum adsorption capacities of DEA-type and EDA-type adsorbents are 1.335 mmol/g and 0.909 mmol/g, which are not much different from the experimental test of 1.310 mmol/g and 0.846 mmol/g. Furthermore, as can be seen in conjunction with Figure 4c, the DEA-type adsorbent exhibits higher adsorption capacity compared to the EDA-type adsorbent.

### 3.3. Effect of HCl Concentration on the Adsorption Test

**Figure 6:**
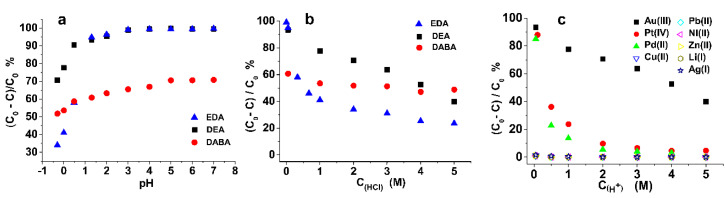
Removal ratio of (**a**) Au(III) with initial concentration of 0.05 mM at various HCl concentration from EDA-type adsorbent, from DEA-type adsorbent and from DABA-type adsorbent; (**b**) removal ratio of all kinds of metal ion from DEA-type adsorbent Au(III), Pt(IV), Pd(II), Cu(II), Pb(II), Ni(II), Zn(II), Li(I), Ag(I); (**c**) Effect of pH on Au(III) adsorption on DEA-, EDA-, and DABA-type adsorbent with 0.01g of adsorbent in 10 mL of 0.05 mM simulate Au(III) solution.

As shown in Figure 6a, the effect of pH variation in adsorption of Au(III) by DEA-, EDA-, and DABA-type adsorbent was studied. The removal ratio of three adsorbents for Au(III) increased with the increment of pH, for DEA and EDA-type adsorbent when pH reaches 3, the adsorption capacity reached the maximum, this result is similar to Figure 6b. Figure 6b shows the removal ratio of Au(III) from EDA-, DEA-, and DABA-type adsorbent at different HCl concentrations from 5 mM to 5 M to investigate the effect of HCl concentration on the adsorption. The removal ratio of three adsorbents for Au(III) decreased with the increment of HCl concentration. Moreover, the trend of decrement obtained from EDA-type adsorbent was extremely notable, while DABA-type adsorbent was inert toward HCl. This could be caused by the ion-exchanged capacity for secondary and tertiary ammonium which could be influenced by HCl in solution while quaternary ammonium could keep a relatively stable capacity.

The adsorption ability of DEA-type adsorbents for some other metal ions including Pt(IV), Pd(II), Cu(II), Pb(II), Ni(II), Zn(II), Li(I) and Ag(I) at different H^+^ concentrations was investigated, as shown in Figure 6c. The percentage adsorption of other precious metal of Pt(IV) and Pd(II) decreased rapidly with the increment of H^+^ concentration. In high concentration of more than 3 M H^+^ the adsorption percentage was almost negligible. Meanwhile, the adsorption for other metal ions is almost zero in the whole experimental range, and this is mainly because the form of Au(III) in acid solution is anionic AuCl_4_^−^ [31]. However, some coexisting metal ions, such as Cu(II), Zn(II), and Ni(II), exhibit electrical neutrality or positively [33,34]. Furthermore, the adsorbent modified by organic amine will be positively charged by protonated in an acidic solution. Based on the above findings, the high selectivity adsorption for Au(III) can be attributed to electrostatic attraction. which indicated that this amine-type adsorbent exhibited excellent selectivity to Au(III) over other metals of Cu(II), Pb(II), Ni(II), Zn(II), Li(I) and Ag(I) in H^+^ solution.

### 3.4. Column Mode Adsorption and Separation of Au(III)

**Figure 7:**
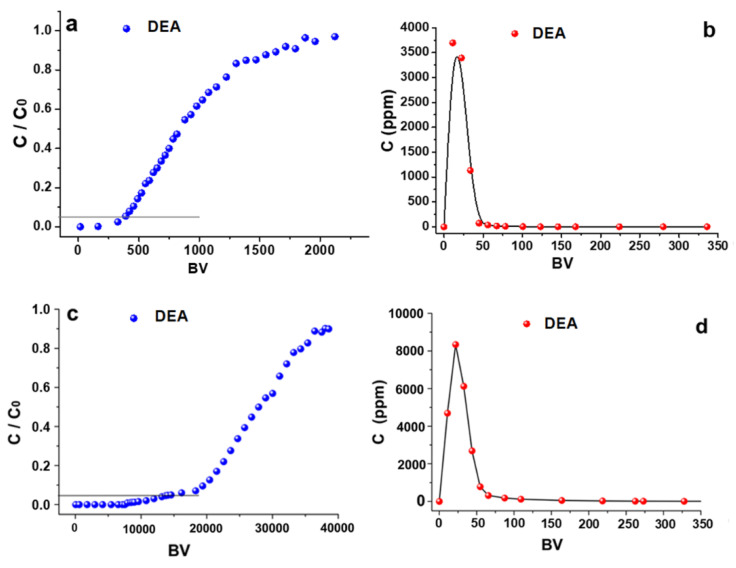
(**a**,**c**) Breakthrough and (**b**,**d**) elution profiles of 0.5 mM Au(III) with 1 M HCl and 0.05 mM Au(III) with 5 mM HCl from a column packed with DEA-type adsorbent, respectively.

**Figure 8:**
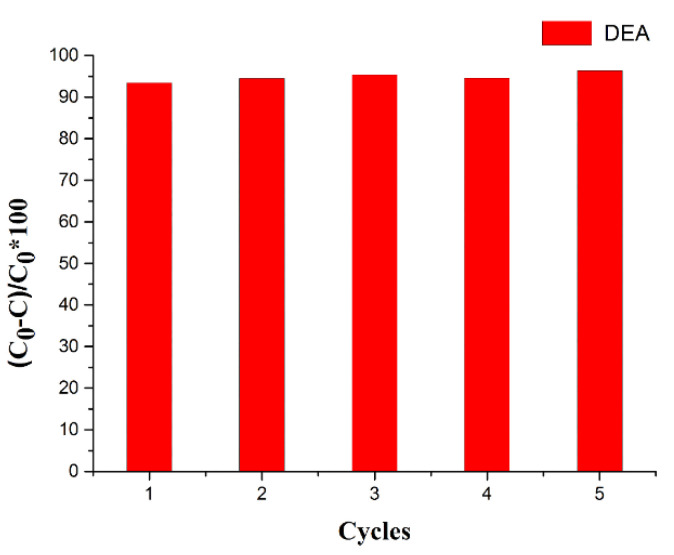
Adsorbed amounts of Au (III) on DEA-type adsorbent in the successive adsorption–regeneration cycles.

Figure 7a,b shows the typical breakthrough curves of Au(III) solution. The abscissa is the dimensionless effluent volume defined by the volume ratio of the effluent to the absorbent. The ordinate is the concentration ratio of the effluent to the feed, and when the outflow concentration reaches 5% of the initial concentration, the corresponding point of the corresponding breakthrough curve is called the breakthrough point. The breakthrough curves were observed by pumping 0.5 mM Au(III) solution with 1 mM HCl into the DEA-type column with a flow rate of 1000 h^−1^ in space velocity (SV). The breakthrough point was observed after 500 BV (Figure 7a). From the area of the breakthrough curve, the equilibrium adsorption capacity (EC) was evaluated as 1.07 mmol/g. When adsorption was carried out with 0.05 mM Au(III) solution with 5 mM HCl with a flow rate of SV 1000 h^−1^. The breakthrough point was observed after 15,000 BV (Figure 7c). Equilibrium adsorption capacity was evaluated as 3.23 mmol/g of the adsorbent. It should be noted that the value is higher than that of the functional group density (2.9 mmol/g), which indicated that aggregation of Au not only depended on the ion-exchange capacity but also reducing capacity of the adsorbent. The reduction of Au(III) into its nano-size particles was clearly observed during the adsorption process as the packed column gradually changed color to dark violet (Figure 10a). After adsorption, Au was easily eluted by a mixture of 0.2 M thiourea solution with 0.01 M HCl. The adsorbed Au was almost completely eluted after 100 BV at a flow rate of 1000 h^−1^ SV (Figure 7b,d). Compared with conventional adsorbents the adsorption capacity has been dramatically improved [35,36]. The extremely rapid removal of Au(III) with a high flow rate of 1000 h^−1^ SV indicated easy regeneration and excellent reusability of this DEA-type materials. Figure 1h,i showed the SEM image of the EDA- and DEA-type adsorbent after adsorption and elution. The surface images indicated morphology of the adsorbent seldom changed during the adsorption or elution process, which reveals reusage of this amine-type adsorbent. Besides, the reusability of DEA-type adsorbent was evaluated. As shown in Figure 8, in the condition of the concentration of gold is 0.05 mM at 5 mM HCl. After five cycles of adsorption, the removal ratio of DEA-type adsorbent can still maintain more than 90%.

**Figure 9:**
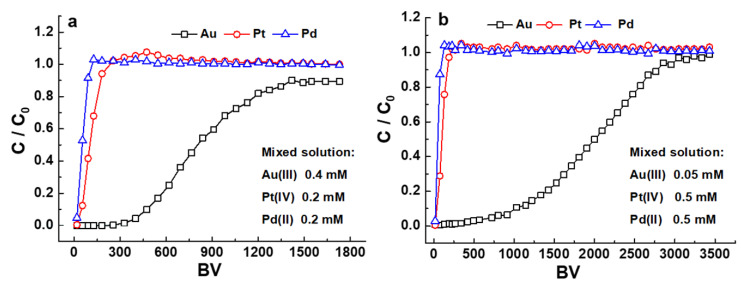
Breakthrough curves of Au(III), Pt(IV) and Pd(II) from (**a**) 0.4 mM Au(III), 0.2 mM Pt(IV) and 0.2 mM Pd(II) mixed solutions; (**b**) 0.05 mM Au(III), 0.5 mM Pt(IV) and 0.5 mM Pd(II) mixed solutions, obtained by DEA-type adsorbent packed in a column.

A breakthrough experiment was conducted to extract Au(III) with different concentrations from its mixed solution containing Pd(II) and Pt(IV) by DEA-type adsorbent packed in a column. As was shown in Figure 9, it could be observed that Pt(IV) and Pd(II) broke through very fast at the start of the process while the breakthrough of Au(III) took place after 400 and 800 BV, in high concentration feed solution (Figure 9a) and low concentration feed solution (Figure 9b) of Au(III), respectively. The possible reason is that in acidic solutions, Au(III), Pt(IV) and Pd(II) exist in the form of AuCl_4_^−^, PtCl_6_^2−^ and PdCl_4_^2−^, respectively [37,38,39]. In the early stage of adsorption, the adsorption sites can combine with Au(III), Pt(IV) and Pd(II). With adsorption continues, because of the limited adsorption sites and the high affinity between Au(III) with amine groups, some of the adsorbed Pt(IV) and Pd(II) ions will be replaced by Au(III) [40]. Moreover, in previous studies, it was also found that the combination of Pd(II) and adsorption sites was unstable [41]. This means DEA-type adsorbent exhibited selective adsorption of Au(III) not only from basic metals but also from other precious metals such as Pd(II) and Pt(IV).

### 3.5. Reduction of Au(III)

**Figure 10:**
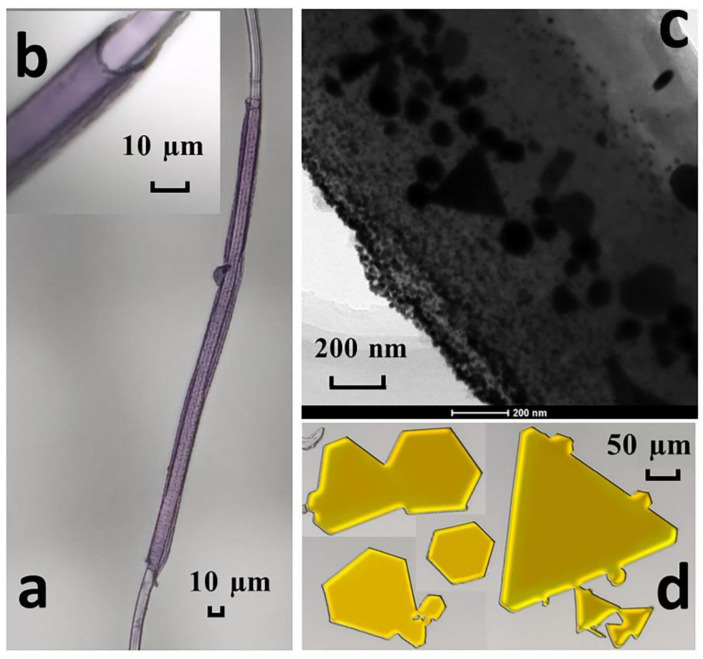
After adsorption test from 0.05 mM Au(III) in 5 mM HCl, (**a**,**b**) microscope image of the DEA-type fiber; (**c**) TEM image of the DEA-type fiber loaded with nanoparticles; (**d**) microscope images of nanocrystals of gold after 0.5 mM Au(III) adsorption in 1 M HCl.

**Figure 11:**
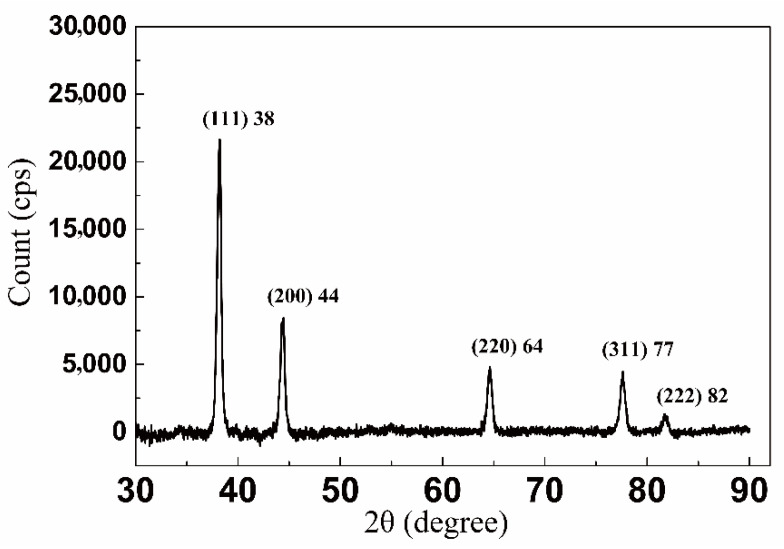
XRD spectra of the DEA-type adsorbent loaded with gold.

The formation of gold during the adsorption process of Au(III) was observed in Figure 10. It is well known that gold nanostructures display a very intense color because of their surface plasmon resonance. After the removal of Au(III) from its solution with 5 mM HCl, the color of the amine-type adsorbent gradually changed from light to dark violet (Figure 10a,b). TEM and XRD analysis show the major products are spherical gold nanoparticles (Figure 10c and Figure 11). However, no precipitation was observed in the solution, which means the nanoparticles aggregated in the adsorbent. The microscopy image in Figure 10a,b indicated that gold aggregation only occurred in the outer layer of PE fiber. Powder XRD analysis gave us further information of the structures of the microplates. As shown in Figure 11, the XRD peaks appeared at (111), (200), (220), and (311), indicating gold with a face-centred cubic (fcc) structure (JCPDS card no. 04-0783). The (111) peak in the gold plate was the strongest among those in these three kinds of gold nanoparticle morphologies. Taking account to the aqueous chemistry of anionic chloride complexes of Au(III), AuCl_4_^−^ is a powerful oxidizing agent in solution with standard reduction potentials (E^0^) of 1.0 V. According to reports, polymers carrying amino groups have the role of reducing agents and stabilizers. Therefore, the in-situ reduction of adsorbed gold can occur through the following equations [42,43].

(9) $${\mathrm{AuCl}}_{4}^{-}{\;+\;3e}^{-}\to \;\mathrm{Au}\left(0\right){\;+\;4\mathrm{Cl}}^{-}\;{\;E}^{0\;}+1.0\;(V)$$

(10) $${2\mathrm{R-}\mathrm{CH}}_{2}{\mathrm{-NR}}_{2}{\;+\;\mathrm{AuCl}}_{4}^{-}\;\to {2R-\mathrm{CH}\;=\;N}^{+}R_{2}+\mathrm{Au}↓{+2H}^{+}{+4\mathrm{Cl}}^{-}\;$$

In the solution with 1 M HCl, plate-like Au particles and product aggregation were observed in the surface of the adsorbent and the bottom of the container. The products displayed a wonderful golden hue with equilateral, truncated triangular and hexagonal shapes with the length of more than 100 µm (Figure 10d). The large size of the Au can easily be separated from the adsorbent compared with the nano-size particle, which might be beneficial for the recovery of Au. The formation mechanism for nano and micro gold particles was widely studied [44,45,46,47,48]. A hypothetic growth mechanism of gold nanocrystal contains two processes: (i) the formation of gold atoms or small clusters in the amino group domain as the nascent crystal nuclei; (ii) anisotropic growth from these nuclei; nano-size particles would be loaded in the fiber, while a micro-sized plate resulted in precipitation from the solution. Influence factor including Au(III) concentration, HCl concentration and temperature affected the formation process and size of gold, which would be another interesting topic. However, considering the space constraints, the detailed explanation is omitted in this paper.

## 4. Conclusions

Varieties of amine-type adsorbents synthesized via radiation-induced graft polymerization were investigated to recovering Au(III) from aqueous solution. The SEM, FTIR and XPS were used to analyze the morphology, synthesis of materials and adsorption performance on the materials. Sorption isotherm and sorption kinetics suggested that the adsorption behavior was fitted well with the Langmuir isotherm model and pseudo-second-order kinetic model. In the co-existing metal ions adsorption experiment, due to the electrostatic repulsion, DEA-type adsorbent was inert toward other metal ions including Cu(II), Pb(II), Ni(II), Zn(II), and Li(I) within the fixed concentration range of HCl, which exhibited DEA-Type adsorbent excellent selectivity. Meanwhile, in the column adsorption test, DEA-type adsorbent also exhibited high selectivity and rapid extraction for Au(III) from its mixed solution with Pt(IV) and Pd(II). After adsorption, the reduction of Au(III) to its elemental form was investigated. Aggregation of nano-size Au particles and micro-size Au plates were observed in different conditions. Based on the excellent selective adsorption, reduction and elution for Au (III), DEA-type NF would be applied for the selective and effective gold recovery from aqueous solution.

## Figures and Tables

**Table 1:** Information of functional groups.

Organic Amine	Structure	Type of Ammonium	Functional Group Density (mmol/g)
EDA	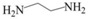	Secondary	2.9
DETA	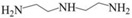	Secondary, Tertiary	1.7
TETA	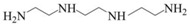	Secondary, Tertiary	1.6
DEA	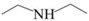	Tertiary	2.2
TAEA	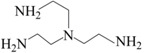	Secondary, Tertiary, Quaternary	1.5
DABA	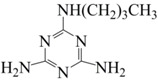	Secondary, Tertiary	0.2

**Table 2:** Kinetic parameters of the sorption for Au(III) by amine-type adsorbent at different conditions.

Solution		Adsorbent					
Au(III) (mM)	HCl (M)	EDA	DETA	TETA	DEA	TAEA	DABA
		k_2_ (kg mol^−1^ s^−1^)					
0.05	5 × 10^−3^	0.03055	0.02454	0.02845	0.02419	0.01387	0.01313
0.5	1	0.05903	0.05885	0.05321	0.00357	0.02324	0.02615

**Table 3:** Langmuir and Freundlich Isotherm Parameters for Au(III) onto DEA-type and EDA-type adsorbent.

Sample	Langmuir		
	Q_max_ (mmol/g)	K_L_ (L/mmol)	R^2^
DEA	1.335	4.179	0.999
EDA	0.909	1.663	0.998
